# On the in Vitro Biocompatibility Testing of Bioactive Glasses

**DOI:** 10.3390/ma13081816

**Published:** 2020-04-12

**Authors:** Devis Bellucci, Elena Veronesi, Massimo Dominici, Valeria Cannillo

**Affiliations:** 1 Department of Engineering “Enzo Ferrari”, University of Modena and Reggio Emilia, Via P. Vivarelli 10, 41125 Modena, Italy; valeria@unimore.it; 2 Department of Medical and Surgical Sciences for Children & Adults, University of Modena and Reggio Emilia, Hospital of Modena, Via del Pozzo 71, 41125 Modena, Italy; elena.veronesi@unimore.it (E.V.); massimo.dominici@unimore.it (M.D.); 3 Scientific and Technological Park of Medicine “Mario Veronesi”, via 29 Maggio 6, 41037 Mirandola, Italy

**Keywords:** human bone marrow mesenchymal stem cells, 3D cellular model, tissue engineering, bioactive glass, 45S5 Bioglass^®^

## Abstract

In this work, a new 3D cellular model—based on human bone marrow mesenchymal stem cells (BM–MSCs)—was used for the first time to test the 45S5 Bioglass^®^ (45S5). Such a model, previously used to evaluate the biologic performance of two novel bioactive glasses, suggested out the regenerative potential of such materials. In fact, BM–MSCs were able both to adhere and colonize the biomaterials, as well as differentiate toward osteoblasts—even in absence of specific growth factors. Surprisingly, BM–MSCs were not able to colonize 45S5 granules (almost no adhesion and/or colonization was observed), and thus, were not capable of providing any osteogenic differentiation. Therefore, the model seems to suggest that the two novel bioactive glasses have a better biologic performance than 45S5. If this hypothesis is confirmed also by in vivo tests, the 3D model may become a predictive tool for discriminating between different potential bioactive materials by comparatively evaluating them, and preliminarily selecting the best ones in relation to their biocompatibility potential—before proceeding with further experiments in vivo. This approach could favor the reduction of costs and time of pre-clinical and clinical trials.

## 1. Introduction

Every year, all over the world, 20 million patients are affected by bone defects [1]. In particular, osteosarcoma, osteoarthritis and osteoporosis are the principal pathologies associated to the musculoskeletal system, along with cancer and tissue loss due to bone fractures. The most compromised clinical cases require surgery and it is estimated that each year millions of people need a surgical procedure aimed to replace lost or missing bone [2].

Nowadays, clinical practice is mainly based on the use of autografts or allografts. In autografts, bone tissue is harvested from the patients’ body itself (from another site): even if this procedure does not show an immunogenic rejection, autografts have several drawbacks, such as inadequate amount of bone tissue, in particular in children, donor site morbidity and pain and additional surgical time [3]. On the other hand, allograft tissue is taken from an individual other than the one receiving the graft, however there is a potential risk of infections transmission [3]. To overcome these drawbacks, synthetic bone grafts have been developed to offer a valid alternative.

Among others, bioactive glasses [4,5,6] have gained a lot of attention because of their attractive properties in terms of ability to bond to bone. The 45S5 Bioglass^®^ (45S5) [4,5,6] opened new and intriguing scenarios in the field of bone regeneration, especially due to its osteoconductive and osteoinductive ability.

In recent years, many other bioactive glasses have been developed and investigated—especially those containing the so-called therapeutic ions [7]. Such new bioactive glasses may have very interesting properties; however, these novel and promising materials require extensive research before they can be put in the market. In vivo tests in animals remain necessary in the biomaterials field, but it is desirable to reduce the number of animals sacrificed and refine the methods for biologic assessment, in accordance with the 3R rule (replacement, reduction, refinement). Whenever possible, experiments on live animals should be replaced, at least in part, by predictive and reliable in vitro tests.

Recently, an innovative 3D cellular model, able to reproduce the potential clinical application of a given material, was used for the first time to investigate novel bioactive glasses, enriched with strontium and magnesium, namely BGMS10 [8] and Bio_MS [9]. The compositions of such glasses in mol% were respectively: 2.3 Na_2_O; 2.3 K_2_O; 25.6 CaO; 10.0 MgO; 10.0 SrO; 2.6 P_2_O_5_; 47.2 SiO_2_ [8] and 5.0 Na_2_O; 31.3 CaO; 5.0 MgO; 10.0 SrO; 2.6 P_2_O_5_; 46.1 SiO_2_ [9]. The tests were performed in a 3D environment specifically designed to simulate the real environment in the human body; in other words, the operating theatre. The performance of bioactive glasses in such 3D cellular models was evaluated by using human bone marrow mesenchymal stem cells (BM–MSCs). The results pointed out that the two bioactive glasses supported BM–MSCs adhesion, proliferation and bone differentiation [8,9]. In particular, the model showed that granules of bioactive glass Bio_MS were able to stimulate the osteogenic differentiation of BM–MSCs, even without proper growth factors, thus revealing the osteoinductivity and the regenerative potential of the material [9]. Since the stem cells used were human, the model was expected to provide realistic and useful indications in view of in vivo applications.

The main goal of this paper is to test, for the first time, the “gold standard” 45S5 by means of the innovative 3D cellular model, with the double aim of: (i) validating such model with reference to the well-known 45S5, for which a lot of results are available in the literature, also in terms of in vivo experiments; (ii) comparing the performance of the two novel bioactive glasses [8,9] with the standard 45S5. In fact, since for the 45S5 in vivo experiments are available, the comparison of the output of the present model with literature data could be useful to assess the viability of such methodology and its reliability.

## 2. Materials and Methods

### 2.1. Preparation of Bioactive Glasses

Bioactive glasses were produced by a classical melt-quenching method, as previously described [8,9]. Briefly, commercial raw powders were melted at 1450 °C for 1 h in platinum crucibles (thermal cycle used: from room temperature to 1100 °C at 10 °C/min; an isothermal step for 1 h at 1100 °C; from 1100 °C to 1450 °C at 10 °C/min). Subsequently, the molten glasses were quenched into water in to obtain glass frits, which were dried for 24 h at 110 °C, ground and then sieved to produce granules (grain size: 100–500 μm, as in [9]).

### 2.2. Biologic Characterization

The 3D cellular model, used to mimic the potential clinical application of a given biomaterial, was fully described in previous works [8,9] and is here only briefly summarized. Such model used human bone marrow mesenchymal stem cells, which were isolated from bone marrow of 3 healthy donors and then expanded ex vivo. Granules of the bioactive glass (here 45S5) were sterilized with UV light for two hours. Then, dry granules were loaded in a multi-well and used for cell seeding, without rinsing or washing. The suitable cell density, defined as the ratio of the number of cells for mg of bioglass, was set equal to 3000/mg (in 200 µl of culture medium), according to [8,9].

The culture medium was composed of α-minimum essential medium (MEM) without nucleosides (Gibco Invitrogen), supplemented with 8% platelet lisate, 1% L-Glutamine (Gibco Invitrogen, Paisley, UK), 1 UI/mL heparin (Sigma-Aldrich, Milano, Italy), 1% Penicillin-Streptomycin, (Gibco). Such culture medium was refreshed every 2–3 days.

BM–MSCs were incubated in proper conditions (5% CO_2_, 37 °C). Crystal violet staining was performed at 1 h, 4 days, 7 days and 14 days post seeding, in order to visualize the cells that adhered to the bioactive glass. Photomicrographs were acquired using a microscope AxioZoom V.16 (Zeiss) equipped with ZEN Pro software. 

## 3. Results and Discussion

The 3D cellular model was set up to investigate 45S5 in order to verify that the material is suitable for its intended use, i.e., bone regeneration.

**Figure 1:**
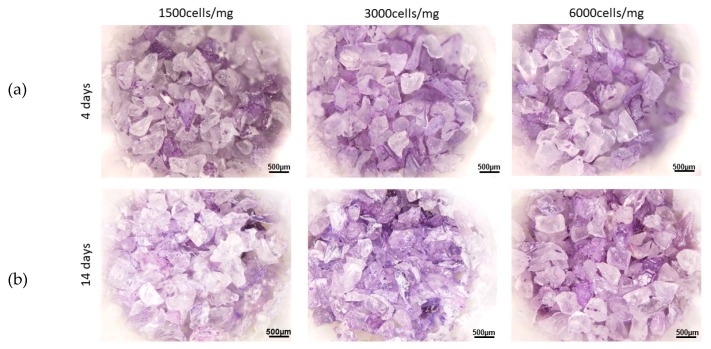
Crystal violet staining of human bone marrow mesenchymal stem cells seeded on 45S5 at 4 days (**a**) and 14 days (**b**) after seeding, with 3 different cells densities (1500/mg, 3000/mg and 6000/mg).

BM–MSCs were seeded in a proper volume of culture medium (200 µl) on dry 45S5 granules, in the ratio of 3000 units for each mg of bioglass. The cells were then observed after 1 h; 4, 7 and 14 days. Surprisingly, both at 1 h post seeding and after 4 days, few adherent BM–MSCs were observed, as reported in Figure 1. After 7 days, just a few zones resulted positive to the crystal violet staining; even after 14 days cells adhesion was not encouraging. Since the results obtained at 3000/mg were not satisfactory, other two cell densities were tested according to the previous work [8], namely 1500/mg and 6000/mg. However, none of the densities gave positive results, as shown in Figure 1 as well.

**Figure 2:**
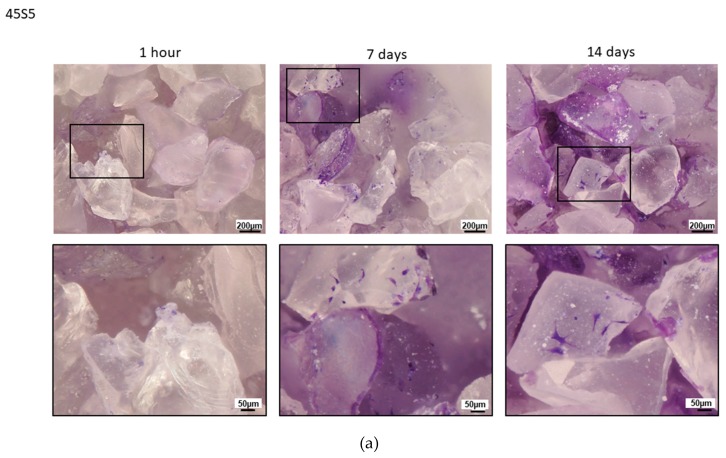

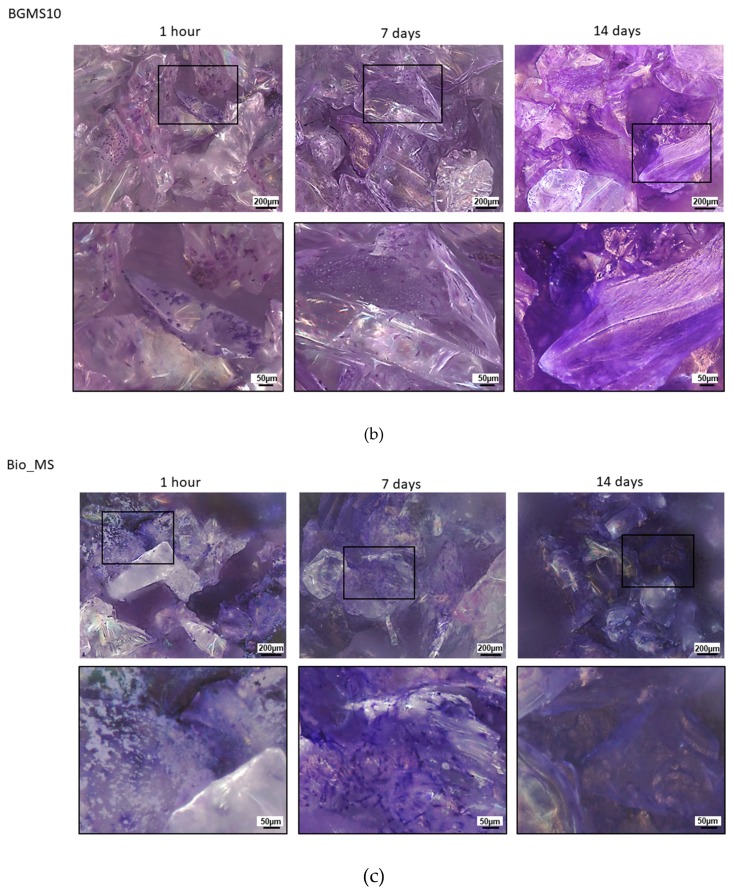
Comparison between 45S5 (**a**), BGMS10 (**b**) and Bio_MS (**c**): crystal violet staining of BM–MSCs seeded on 45S5 (**a**), BGMS10 (**b**) and Bio_MS (**c**) at 1 h, 7 days and 14 days after seeding (cells density: 3000/mg).

To better understand the behavior of 45S5, a comparison was made with the other two bioactive glasses (Figure 2), tested with the same protocol and discussed in previous works [8,9]. While for both BGMS10 and Bio_MS it was possible to observe a greater amount of adherent cells at 1 h post seed, and after 7–14 days BGMS10 and Bio_MS resulted fully colonized, in 45S5 almost no adhesion or colonization of BM–MSCs was observed. Thus, it was not possible to perform a bone differentiation assay.

This behavior of 45S5 was completely unexpected; in fact, even if it is often reported in the literature that such bioglass has a good performance both in vitro and in vivo [10,11], in the present model BM–MSCs were not able to adhere and colonize 45S5. In [11], the authors observed the osteogenic differentiation of two different types of mesenchymal stem cells seeded on 45S5. Anyway, before seeding, the bioglass was incubated for 2 weeks in a cell culture medium, and the medium was refreshed every day (probably in order to avoid high and abrupt variations of pH values in the culture medium due to the fast ion release from the glass). In the present study, instead, mesenchymal stem cells were not able to give any osteogenic differentiation. Probably, this fact can be attributed to both (1) the specific compositions of the novel BGMS10 and Bio_MS, which looked very promising in terms of biologic responsiveness, in particular thanks to the presence of specific ions such as strontium [12] and (2) the pH rising, due to the ions release from 45S5, once in contact with physiological media; on the contrary, the slower ion leaching from both BGMS10 and Bio_MS glasses resulted in a pH of the culture medium near to physiological levels, as discussed in [8,9]. The phenomenon of pH increase for 45S5 and, more in general, for bioactive glasses due to their high reactivity and ionic release, once in contact with water-based solutions, is well documented in the literature and makes difficult to perform cell culture investigations. This fact may be at least partially responsible of the behavior reported in Figure 2, as a basic pH is highly unfavorable to cells survival. The technique use for example in [11]—i.e., the preconditioning of the bioglass in cell culture medium or in a simulated body fluid solution for several days before cell seeding—is likely to overcome this problem. A similar technique was used also in another work [13], where 45S5 scaffolds were soaked in SBF for 22 days, changing the solution every day, prior to cell seeding. Although several pre-conditioning treatments have been proposed to tackle the pH increase issue with bioactive glasses [14], it would be of fundamental importance to create the most similar situation to the physiological one of the human bone tissue, and, moreover, to design novel bioactive glasses with a reactivity tailored to the physiological environment; in this context, the 3D model here discussed mimics the in vivo micro-environment, i.e., the operating theatre, where the bioactive materials are directly delivered. For these reasons, the materials were not preconditioned before cell seeding and they were used as prepared (after sterilization).

Nonetheless, in vivo 45S5 seems to perform well (and it is currently sold in the market). Probably the present 3D cellular model should be further improved to account for in culture dynamic conditions (and pH control).

Anyways, the scope of the present study was to set up a model that could be useful to discriminate between different potential bioactive glasses/materials. If this task is achieved, the model could be used as a predictive tool: in fact, if BGMS10 and Bio_MS perform better than 45S5 in vivo, this means that the model is able to identify bioactive glass compositions which can be very effective for tissue engineering and bone regeneration. Thus, the model could be used to comparatively evaluate different materials and to preliminary select the best ones in relation to the biologic performance, before proceeding with further experiments in vivo. This strategy could in turn reduce costs and time of such in vivo experimentations and of pre-clinical and clinical trials.

In conclusion, the model presented here is a novel approach which has two inherent advantages: (a) it is more similar to the physiological situation of human bone tissue; (b) it seems to have more stringent requirements than conventional cell culture approaches and thus the method is potentially useful to identify new bioactive materials with a tailored reactivity, which do not cause an excessively high and unfavorable variation in pH.

The next step of the present research will tackle the problem by in vivo tests (in animals) to verify if BGMS10 and Bio_MS perform better than 45S5, thus confirming their optimal biologic responsiveness. If this happens, two important points follow: (i) the reliability of the 3D cellular model, at least in terms of comparative evaluation, is confirmed; (ii) the two novel bioactive glasses, BGMS10 and especially Bio_MS, are really promising.

## 4. Conclusions

In this work, an innovative 3D cellular model has been used to test for the first time the 45S5 Bioglass^®^, which is considered as “gold standard” and a reference among bio glasses. Previously, two innovative bioactive glasses have been investigated with the same model and gave very positive results for the intended use, i.e., bone regeneration; the aim of the present research was to test the well-known 45S5, to set a reference. This is very important in the light of having a reliable in vitro model that could reduce the burden of in vivo experiments.

Unexpectedly, 45S5 could not be colonized by human bone marrow mesenchymal stem cells and thus it was not even possible to perform a bone differentiation assay. Therefore, the model seems to suggest that the two previously investigated materials, i.e., BGMS10 [8] and Bio_MS [9], behave better than 45S5 in terms of overall biocompatibility and biologic performance. This fact can be attributed to their specific compositions, which looked very promising in terms of biologic responsiveness [12] and to their ion release which is slower to that from 45S5, once in contact with physiological media; in particular, the ion leaching from both BGMS10 and Bio_MS glasses resulted in a pH of the culture medium near to physiological levels, as discussed in [8,9]. Moreover, such novel bioactive glasses can be readily used as prepared, without any preconditioning, rinsing or washing in cell culture medium or physiological solutions.

The next step of the research will be to confirm this output by means of in vivo experiments (in animals); if this is verified, (i) both BGMS10 and Bio_MS have superior properties compared to 45S5 and (ii) the model is reliable and could be considered as an important preliminary screening for new bioactive materials.
